# A Chatbot-Delivered Stress Management Coaching for Students (MISHA App): Pilot Randomized Controlled Trial

**DOI:** 10.2196/54945

**Published:** 2024-06-26

**Authors:** Sandra Ulrich, Natascha Lienhard, Hansjörg Künzli, Tobias Kowatsch

**Affiliations:** 1 School of Applied Psychology Zurich University of Applied Sciences Zurich Switzerland; 2 Institute for Implementation Science in Health Care University of Zurich Zurich Switzerland; 3 School of Medicine University of St. Gallen St.Gallen Switzerland; 4 Centre for Digital Health Interventions Department of Management, Technology and Economics ETH Zurich Zurich Switzerland

**Keywords:** conversational agent, mobile health, mHealth, smartphone, stress management, lifestyle, behavior change, coaching, mobile phone

## Abstract

**Background:**

Globally, students face increasing mental health challenges, including elevated stress levels and declining well-being, leading to academic performance issues and mental health disorders. However, due to stigma and symptom underestimation, students rarely seek effective stress management solutions. Conversational agents in the health sector have shown promise in reducing stress, depression, and anxiety. Nevertheless, research on their effectiveness for students with stress remains limited.

**Objective:**

This study aims to develop a conversational agent–delivered stress management coaching intervention for students called MISHA and to evaluate its effectiveness, engagement, and acceptance.

**Methods:**

In an unblinded randomized controlled trial, Swiss students experiencing stress were recruited on the web. Using a 1:1 randomization ratio, participants (N=140) were allocated to either the intervention or waitlist control group. Treatment effectiveness on changes in the primary outcome, that is, perceived stress, and secondary outcomes, including depression, anxiety, psychosomatic symptoms, and active coping, were self-assessed and evaluated using ANOVA for repeated measure and general estimating equations.

**Results:**

The per-protocol analysis revealed evidence for improvement of stress, depression, and somatic symptoms with medium effect sizes (Cohen *d*=−0.36 to Cohen *d*=−0.60), while anxiety and active coping did not change (Cohen *d*=−0.29 and Cohen *d*=0.13). In the intention-to-treat analysis, similar results were found, indicating reduced stress (β estimate=−0.13, 95% CI −0.20 to −0.05; *P*<.001), depressive symptoms (β estimate=−0.23, 95% CI −0.38 to −0.08; *P*=.003), and psychosomatic symptoms (β estimate=−0.16, 95% CI −0.27 to −0.06; *P*=.003), while anxiety and active coping did not change. Overall, 60% (42/70) of the participants in the intervention group completed the coaching by completing the postintervention survey. They particularly appreciated the quality, quantity, credibility, and visual representation of information. While individual customization was rated the lowest, the target group fitting was perceived as high.

**Conclusions:**

Findings indicate that MISHA is feasible, acceptable, and effective in reducing perceived stress among students in Switzerland. Future research is needed with different populations, for example, in students with high stress levels or compared to active controls.

**Trial Registration:**

German Clinical Trials Register DRKS 00030004; https://drks.de/search/en/trial/DRKS00030004

## Introduction

### Background

Stress is rapidly becoming a major issue affecting adults in high-income countries, especially during periods of uncertainty and worry. Chronic stress is closely related to mental illnesses such as anxiety disorders and depression, leading to various symptoms such as sleep disturbances, pain, dizziness, cardiovascular and digestive problems, as well as fatigue [1,2]. Younger individuals, particularly students [3-7], are experiencing a decline in mental health on a global scale [8,9]. Studies indicate that approximately 11% of students experience impairments such as anxiety, depression, exhaustion, and burnout-like symptoms [1,10]. Furthermore, a high level of stress can have a negative impact on academic performance, resulting in changes in study direction, prolonged studies, and even dropout [11,12].

Students encounter distinct challenges during their academic journey, including the need to assimilate a substantial amount of content, effectively manage their time, cope with performance expectations, and handle examination pressure [13]. In addition, the developmentally sensitive period associated with this age group, combined with the academic environment, can contribute to increased stress levels [6]. Furthermore, compared to previous generations, today’s students appear to exhibit lower stress tolerance and inadequate stress coping mechanisms, which further exacerbate the situation [1,14,15]. Notably, a recent study by Ehrentreich et al [16] reported that stress levels among students have increased by nearly 40% due to the impact of the COVID-19 pandemic.

To prevent students from experiencing chronic stress and its long-term effects, the implementation of appropriate prevention programs is crucial. These programs aim to promote students’ self-management and stress management skills, including learning and time management techniques, to help them effectively cope with stress and to counteract increasing stress levels in the target group [10,17]. Studies have demonstrated the positive impact of interventions such as behavioral therapy–based approaches, relaxation and mindfulness exercises, psychoeducation, and time and study management strategies in reducing stress among students [10,18,19]. Typically, evidence-based stress management programs combine psychoeducational sessions with relaxation exercises [20-22]. Importantly, stress management programs should be specifically tailored to the needs of students. By considering the target group’s real-life context, these programs facilitate the transfer of acquired skills into everyday life [23].

Despite the importance of stress management programs for students, successful uptake remains challenging [24]. Unfortunately, individuals experiencing stress often do not make use of stress management techniques for several reasons. These include the fear of being stigmatized [25], underestimation of the impact of stress, limited availability of therapy options, and high cost, particularly for young people in education [26,27].

Low-threshold, mobile health (mHealth) interventions such as smartphone apps could potentially bridge this gap. A meta-analysis by Weisel et al [28] highlighted the advantages of apps, including location and time independence, reduced stigmatization, and low costs [29]. Initial evidence suggests that smartphone apps can effectively reduce perceived stress, distress, depression, and anxiety and improve quality of life, psychological health, well-being, and self-regulation among student populations [30-32]. However, reported disadvantages of digital interventions, such as low adherence, legal concerns, lack of therapist relationship, and arbitrary scheduling, may diminish their effectiveness [29,33].

Conversational agents (CAs), commonly known as chatbots, are designed to simulate humanlike conversations and are increasingly used in clinical and nonclinical settings [34-36]. Initial findings demonstrate the feasibility, acceptance, and effectiveness of CAs in various health domains [37,38], including promoting physical activity [39]; managing pain [40]; reducing substance abuse [41,42]; improving depression, distress, and stress [43]; enhancing general wellness and pain [44]; and facilitating self-adherence and psychoeducation [38]. Although large language model (LLM)–based CAs have recently gained increasing attention [45], they are still subject to basic research in computer science because of several severe shortcomings, such as hallucinations and nonconscious bias, among others [46]. Therefore, LLM-based CAs are not yet appropriate for safe and ethical delivery of several-week health interventions [47]. Hence, we decided to implement an established, safe, and transparent approach to using CAs and used a rule-based CA [39,40,48-51].

Studies investigating the effectiveness of stress management interventions delivered by a CA specifically tailored to the needs of students are still lacking. While recent studies have explored interventions such as Stressbot, developed with Meta’s Messenger (Meta Platforms, Inc) and CA Atena, accessible via Telegram messaging app (developed by the Digital Health Lab at Fondazione Bruno Kessler FBK research center), their focus has been limited to short-term outcomes or specific topics. For instance, while Stressbot aimed to reinforce coping self-efficacy, its intervention period was only 7 days [52]. Similarly, CA Aetna’s positive psychology and cognitive behavioral approaches with a tailored focus on the unique needs of the COVID-19 pandemic rather than the life context of students led to inconclusive outcomes regarding anxiety and stress reduction [53]. Furthermore, a previous study evaluating an artificial intelligence (AI)–based chatbot that provided self-help interventions for students to reduce depression lacked detailed descriptions of evidence-based intervention designs, leaving uncertainty about the elements implemented [54]. However, evidence-based design is vital in developing CA-based coaching intervention programs [34] and stress management interventions for specific groups such as students [23]. To our knowledge, there is no study describing the development and evaluation of the effectiveness of a CA-delivered stress management coaching program lasting several weeks and adapted to the specific context of students in their everyday lives.

Consequently, we have developed an evidence-based, scalable, and CA-delivered stress management coaching intervention for students called MISHA. It combines the following components: (1) providing psychoeducation about stress, mindfulness, and relaxation; (2) fostering participant motivation for self-reflection on stress and stress reactions; and (3) guiding participants in the regular practice of mindfulness and relaxation techniques. This comprehensive approach addresses key aspects of stress management, including knowledge acquisition, self-reflection, and practical application of mindfulness and relaxation techniques [19,55]. By focusing on these evidence-based intervention components, MISHA aims to empower students with effective tools and strategies to reduce stress and its long-term effects.

### Objectives

The goal of this pilot study was twofold: (1) to develop a scalable, evidence-based coaching intervention specifically designed for students and delivered via a CA and (2) to assess the coaching intervention’s effectiveness, engagement, and acceptance.

## Methods

### Intervention

#### App Development

**Figure 1:**
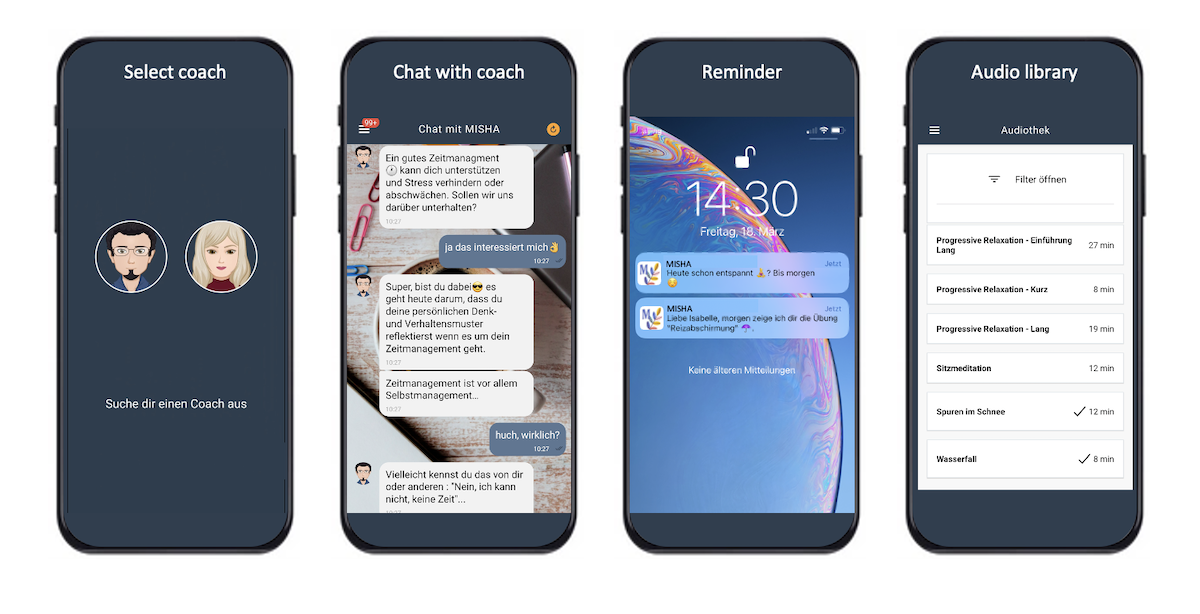
Screenshots of the MISHA app (coach selection, chat interface, reminder, and audio library). Translation from German to English, screenshot Select coach: "Choose a coach"; screenshot Chat with coach: "Effective time management can support you and prevent or reduce stress. Shall we discuss this?", "Yes, I’m interested.", "Great, you’re on board. Today, we’ll focus on reflecting on your personal thought and behavior patterns related to time management. Remember, time management is primarily self-management.", "Really?", "Perhaps you’ve experienced this yourself or observed it in others…"; screenshot Reminder: "Have you relaxed today? See you tomorrow", "Dear Isabelle, tomorrow I’ll show you a relaxation exercise”; screenshot Audio library: “Progressive Relaxation - Introduction (long)", "Progressive Relaxation - Brief", "Progressive Relaxation - Extended", "Seated Meditation", "Footprints in the Snow", "Waterfall”.

MISHA was developed in collaboration with the ETH Zurich using the open-source software platform MobileCoach [56], designed for rule-based digital health interventions [48,57-59]. MISHA features a chat-based interface with multimedia elements and regular notifications to engage users. The app includes a chat channel, an audio library with relaxation exercises, psychoeducational illustrations, and frequently asked questions (Figure 1). Communications takes place via predefined but dynamic answer options or by providing free-text input. Study participants were provided with access to a beta version of the MISHA app for Android (Google LLC) devices through Firebase [60] and for iOS (Apple Inc) devices through TestFlight [61].

#### Coaching Concept of MISHA

The intervention concept for MISHA draws inspiration from an effective face-to-face prevention program [62], adapting its content and topics to suit a CA-delivered approach. MISHA’s chat messages and notifications are aligned with the health action process approach (HAPA) model, emphasizing both motivational and volitional processes in behavior change [63].

MISHA integrates evidence-based strategies from cognitive behavioral therapy (CBT), mindfulness, and psychoeducation to provide information about stressors and coping techniques [55,64]. The stress management program includes fundamental elements derived from CBT, such as cognitive restructuring, identification, evaluation, and modification of maladaptive thought patterns [65]. In addition, techniques such as behavioral activation and activity monitoring from CBT were applied to directly support the participants in their desired goals in a collaborative approach. For further details on CBTs and session elements, refer to Multimedia Appendix 1. The overall aim is to empower participants to reflect on their daily stressors and effectively manage their stress with new coping techniques.

#### Coaching Content

MISHA offers a consecutive 12-session coaching program based on the stress management manual by Kaluza [20]. Sessions cover psychoeducation on stress, relaxation techniques, and student-specific topics such as examination anxiety. Topics are personalized, for example, setting goals, individual appointments with the CA, or selecting a CA. Participants can schedule sessions every 2 to 4 days, completing the program in 24 to 54 days (refer to Multimedia Appendix 1 for an overview of sessions and a detailed description of the content). Throughout the coaching, participants receive personalized feedback on the progression of the coaching, motivational reminders, and reminders in case of inactivity (refer to Multimedia Appendix 2 for detailed information on reminders). Personalization on an individual level is essential in promoting trust, engagement, adherence, and effectiveness to digital health interventions [66,67].

### Study Design and Procedure

We conducted an unblinded, 2-armed, pilot randomized controlled trial in a population of university students in Switzerland. Study participants were allocated either to a 4-week to 7-week coaching intervention or to a 40-day waitlist control group. This research project was registered at the German Clinical Trials Register accredited by the World Health Organization (DRKS00030004). The trial was conducted following CONSORT-EHEALTH (Consolidated Standards of Reporting Trials of Electronic and Mobile Health Applications and Online Telehealth) guidelines. No significant content changes were made to the coaching intervention during the study period.

After downloading the MISHA app, participants were greeted and provided with information about the study procedure and coaching program. They were explicitly informed that the app does not serve as a substitute for psychotherapy and were given guidance on where to seek further help if needed. Study information was displayed within the app. To proceed, participants had to provide electronic informed consent by confirming that they had read and understood the study information. Subsequently, inclusion criteria were checked, and participants were directed to the baseline self-assessment at preintervention (time point 1; T1) using the app’s in-built LimeSurvey platform (LimeSurvey Project). The MobileCoach software automatically randomized participants into either the intervention or the waitlist control group by a 1:1 allocation using random numbers (0 to 1), with numbers <0.5 assigned to the intervention group. Participants from the intervention group started the coaching program immediately. Upon program completion (1) by working through all the modules or (2) after 54 intervention days, participants were directed to the postintervention survey (time point 2; T2) before moving to the final goodbye session. During the intervention, further self-reported outcomes (eg, goal achievement) and use data (eg, total minutes spent on in-app relaxation) were gathered.

Participants from the waitlist control group received short weekly chat messages from MISHA, informing them about the remaining duration of their wait and encouraging them to continue their participation in the study. After 40 days of waiting, they were presented with the postintervention survey (T2) and given the opportunity to participate in the coaching program.

There was no human involvement throughout the study; however, participants had the option to contact the study team via email if they encountered technical issues or encountered problems with app download.

### Ethical Considerations

The Cantonal Ethics Committee of Zurich (KEK-ZH, BASEC-Nr. Req-2020-01038) reviewed the research project and confirmed that the study did not fall within the scope of the Human Research Act. All participants gave informed electronic consent by selecting a checkbox before enrolling in the study and were informed about their right to opt out at any time. Their data were deidentified. Participants who completed the postintervention survey had the opportunity to win a voucher worth CHF 200 (US $224.73). In addition, students of applied psychology at Zurich University of Applied Sciences had the opportunity to earn 5 test person hours.

### Recruitment

From October 6, 2021, to the end of October 2021, flyers were distributed via email to students at the University of Zurich, the Zurich University of Teacher Education, University of Applied Sciences Northwestern Switzerland School of Education, the University of Teacher Education in Special Needs Zurich, and the Zurich University of Applied Sciences. In addition, the flyer was posted on Facebook (Meta Platforms, Inc) and LinkedIn (Microsoft Corp). The app could be downloaded via flyer by following a web link. Eligibility was determined within the MISHA app by self-report and included the following: (1) being aged ≥18 years; (2) possession of and basic knowledge in the use of a smartphone; (3) sufficient knowledge of the German language; and (4) being a student at a Swiss university, university of applied sciences, university of teacher education, or college of higher education.

### Outcomes

#### Primary Outcome

To measure the effectiveness of the program, we assessed perceived stress at preintervention (T1) and postintervention (T2) time points using the German version of the Perceived Stress Scale, a self-report questionnaire consisting of 10 items [68]. Participants rated their responses on a scale ranging from 0 (never) to 5 (very often).

#### Secondary Outcomes

We measured secondary outcomes, including depression, anxiety, somatic symptoms, and active coping, at preintervention and postintervention time points by self-report. Multimedia Appendix 3 presents all outcomes and time points.

##### Depression, Anxiety, and Somatic Symptoms

We used the Patient Health Questionnaire Somatic, Anxiety, and Depressive Symptom Scales [69] to detect depressive symptoms, anxiety, and somatic symptoms, which consists of the Patient Health Questionnaire-9 (PHQ-9), Generalized Anxiety Disorder-7, and the Patient Health Questionnaire-15. The PHQ-9 is a 9-item questionnaire assessing depressive symptoms [70]. Participants rate the frequency of each symptom over the past 2 weeks, ranging from 0 (not at all) to 3 (nearly every day). The Generalized Anxiety Disorder-7 is a 7-item questionnaire that measures anxiety symptoms [71]. Participants rate the frequency of each symptom over the past 2 weeks, ranging from 0 (not at all) to 3 (nearly every day). The Patient Health Questionnaire-15 is a 15-item questionnaire measuring psychosomatic symptoms [72]. Participants rate the severity of each symptom over the previous 4 weeks, ranging from 0 (not bothered at all) to 2 (bothered a lot). For this study, items 14 (trouble with sleeping) and 15 (ie, low energy or tiredness) were collected in the PHQ-9 (similar in both questionnaires) but had to be converted according to the manual [73]. By combining these individual components, the PHQ Somatic, Anxiety, and Depressive Symptoms Scales provide a comprehensive assessment of depressive symptoms, anxiety, and somatic symptoms.

##### Active Coping

According to the HAPA model [74], we evaluated participants’ engagement in stress management activities by asking them to rate how often they had actively taken steps to reduce stress in the past 5 days. The question was assessed on a rating scale ranging from 1 (never) to 4 (regularly). This allowed us to understand the participants’ level of proactive involvement in managing their stress.

#### Predictor: Self-Efficacy Expectancy

Various health behavior change models, including the HAPA model [74], consider self-efficacy expectancy to be a key aspect of health behavior change. However, research findings on the impact on stress interventions are mixed [75-77]. To address this, we assessed self-efficacy expectancy using the General Self-Efficacy Scale [78]*.* Before the intervention, participants rated their agreement with statements on their ability to handle tasks effectively on a 4-point Likert scale ranging from 1 (not at all true) to 4 (exactly true). The total score of the General Self-Efficacy Scale ranges from 10 to 40, with higher scores indicating higher self-efficacy.

#### Exploratory

##### Working Alliance

To assess the interaction between participants and MISHA, we used the German version of the Working Alliance Inventory-Short Revised [79] after the intervention. This self-report questionnaire comprises 12 items that capture the quality of the therapeutic relationship and collaboration between participants and the CA via 3 dimensions: goal, task, and bond. Responses were rated with an adapted scale from 1 (I do not agree at all) to 6 (I completely agree) after the intervention.

##### Subjective Stress Expertise and Goal Achievement

Throughout the coaching period, we assessed participants’ goal achievement 3 times (sessions 1, 6, and 11) using a scale of 1 to 10, where 1 referred to the goal as clearly not achieved and 10 referred to the goal as fully achieved. We further measured participants’ stress expertise 3 times (sessions 2, 5, and 13) using a similar scale, ranging from 1 (no idea how stress manifests itself in me) to 10 (I know exactly how I react when under stress).

#### Engagement and Acceptance

The extent to which a participant has to engage with the intervention to derive the maximum benefits is termed intended use [80]. For MISHA, we defined intended use for participants as completing the postintervention assessment, regardless of completing all sessions. This definition was based on the fact that participants may have varied goals and desired outcomes, leading to differences in their use of MISHA’s features, including frequency and duration [81,82]. It also implies that participants do not necessarily need to interact with all available intervention components. Furthermore, participants might discontinue using the intervention upon achieving their personal goals, indicating that nonuse is not due to loss of interest [83,84]. In addition, we ground this approach on the self-determination theory, where autonomy by providing choice is essential [85].

To assess participants’ engagement in the coaching program, we analyzed use data from the intervention group by calculating the ratio of replied conversational turns based on the number of SMS text messages sent by MISHA in relation to SMS text messages replied by participants. Furthermore, we tracked the number of sessions completed by participants and the number of reminders sent to participants in cases of inactivity (ie, if participants stopped interacting during a session). In addition, we tracked the number of minutes of audio files played by participants throughout the intervention.

We evaluated the feasibility and acceptance of MISHA using the user version of the Mobile App Rating Scale (uMARS) [86] after the intervention. The uMARS is a validated questionnaire that assesses the dimensions of engagement, functionality, esthetics, information, perceived quality, and perceived impact. All subscales use a 5-point Likert scale ranging from 1 to 5, where higher scores indicate a more favorable judgment. In this study, 19 items were translated from English to German to assess engagement (eg, entertainment, interest, customization, interactivity, and target group of the app), information (eg, quality of information, quantity of information, visual information, and credibility of source), perceived quality (eg, recommendation, use, payment, and overall rating), and perceived impact (eg, awareness, knowledge, attitudes, behavior change, seeking help, and intention to change). In addition to the uMARS, participants had the opportunity to provide feedback in free text prompted by the following questions: “What did you like most about the MISHA app?” and “What would you improve in the MISHA app?”

### Sample Size Calculation

The sample size was estimated for a generalized estimating equation (GEE) based on a repeated-measure (within-between interaction) ANOVA. A small to medium time by group interaction effect size (Cohen *f*=0.15) for the primary outcome perceived stress due to prior results [87] was expected. The G*Power (Heinrich-Heine-Universität Düsseldorf) analysis [88] revealed that a sample size of 90 participants would be sufficient with a power of 0.80 and a correlation of *r*=0.5 between measurements. Owing to the high percentage of dropouts observed in earlier studies, the target sample was increased to 180 participants [89].

### Data Analysis

Descriptive statistics, independent 2-tailed *t* tests, and chi-square tests were conducted to analyze baseline differences in demographics and outcomes between the intervention and control groups.

In our analysis, we examined the effectiveness of the intervention by assessing changes in the primary outcome perceived stress scores over time within each group (intervention and control) and comparing these changes between groups. We first conducted a per-protocol (PP) analysis, including only participants who completed both surveys. This was done using a repeated-measure ANOVA with perceived stress as the dependent variable, time as the within-subject factor, and group as the between-group factor. Secondary outcomes, including depression, anxiety, psychosomatic symptoms, and active coping, were analyzed accordingly.

In compliance with the CONSORT (Consolidated Standards of Reporting Trials) guidelines, we also conducted an intention-to-treat (ITT) analysis wherein all randomized participants were included, regardless of their adherence to the coaching intervention. This analysis was performed using GEE. In model 1, we conducted an unadjusted evaluation with time (T1 and T2), group (intervention and control), and treatment (group by time interaction) as independent variables, with perceived stress as the dependent variable. The incorporation of time allows the examination of the dependent variable stress over different time points, the incorporation of group allows for comparison of stress between groups, and the interaction between group and time allows for an examination of whether the changes in outcomes over time differ between the intervention and control groups. In model 2, we did an adjusted analysis with the inclusion of the covariate general self-efficacy for the primary outcome perceived stress. The same independent variables were considered as in model 1. Secondary outcomes were evaluated accordingly. A log link function, gamma distribution, and unstructured covariance structure were applied. This modeling approach provided the best fit with the outcomes and allowed us to avoid restrictions on the covariance structure. To reduce the impact of influential observations and outlier effects, we used a robust estimator, which is consistent with standard procedures when using GEE.

Using GEE [90] offered several advantages. First, it allowed us to consider the correlations between the measurement times in longitudinal data, which is important for analyzing repeated measures. In addition, GEE allowed us to include incomplete data sets using an estimating equation to handle missing data. GEEs use all available data and estimate missing outcome values under the assumption of missing completely at random (MCAR). To assess the assumption of MCAR, we conducted the Little MCAR test. Calculations of between-group effect sizes (Cohen *d*) were based on the pooled SD and labeled as small (Cohen *d*=0.2), medium (Cohen *d*=0.5), and large (Cohen *d*=0.8). Furthermore, we explored the potential relation of working alliance and perceived impact on treatment outcomes using a correlation. All statistical analyses were performed using SPSS software (version 28; IBM Corp). We applied qualitative content analysis [91,92] using thematic maps [93] to answer the open-ended questions.

## Results

### Demographics and Baseline Scores

**Figure 2:**
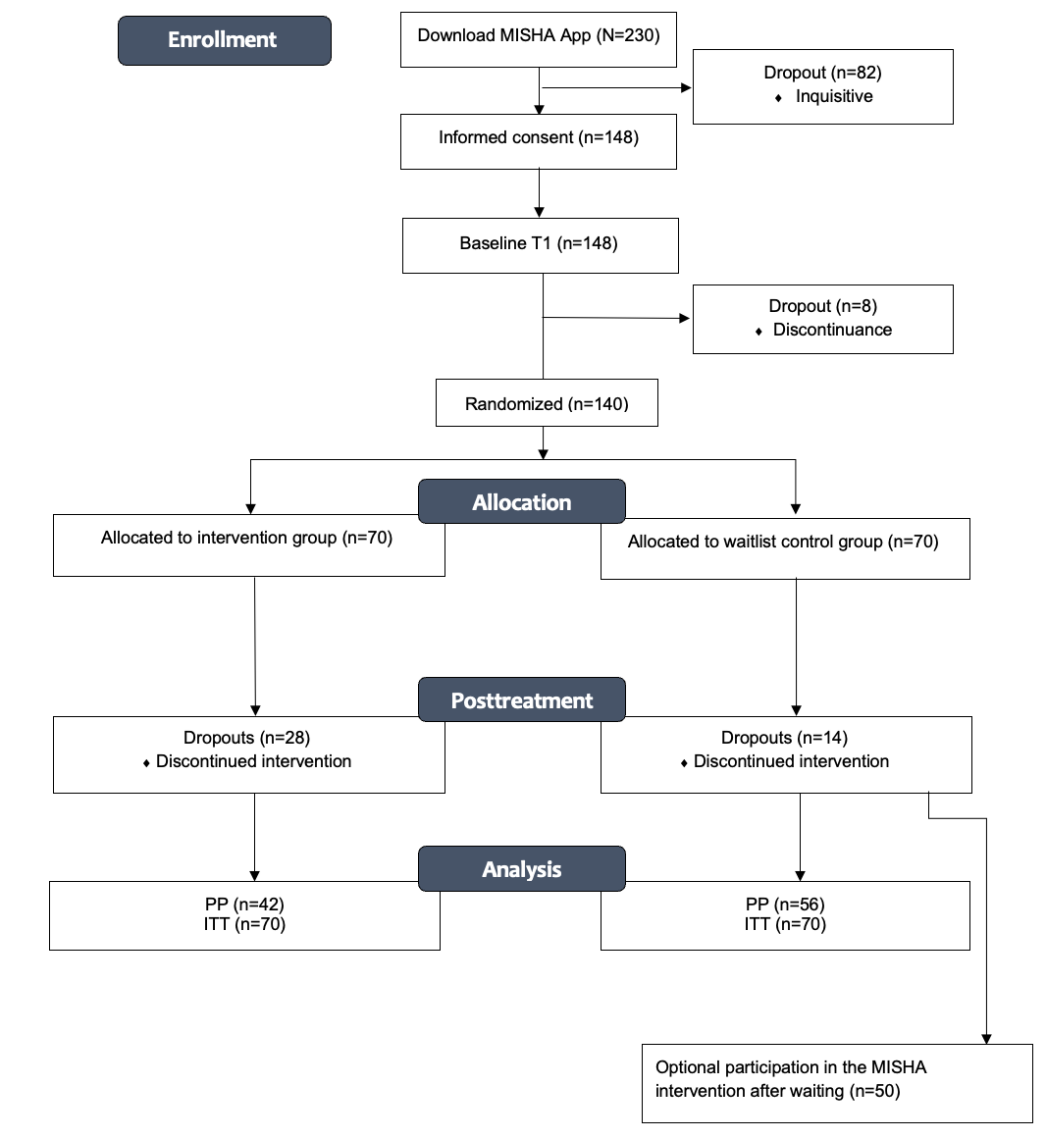
Study flowchart. ITT: intention-to-treat; PP: per-protocol; T1: time point 1.

In total, 230 individuals downloaded the app. Of the 230 individuals, 148 (64.3%) were assessed for eligibility and completed the baseline survey. Before randomization, of the 148 participants, 8 (3.5%) discontinued using the app and 140 (60.9%) were randomized into intervention (70/140, 50%) and waitlist control (70/140, 50%) groups. The complete participant flow is depicted in Figure 2.

Participants had a mean age of 26.71 (SD 6.29) years. While 23.6% (33/140) of the participants identified as men, 73.6% (103/140) as women, and 2.1% (3/140) as nonbinary, 0.7% (1/140) declined to provide information about their gender (Table 1). Regarding relationship status, 59.3% (83/140) of the participants reported being married or in a relationship, while 40.7% (57/140) were single. Regarding educational background, most participants (90/140, 64.3%) had an apprenticeship or vocational or high-school diploma. A substantial proportion of the participants (37/140, 26.4%) had a university degree at the bachelor level or higher vocational education or training, while 8.6% (12/140) had other qualifications. Regarding their field of study, most participants (131/140, 93.6%) were studying at a university of applied sciences or university, while 5% (7/140) were studying at other institutions. The participants had a degree in (applied) psychology (124/140, 88.6%), social sciences (6/140, 4.4%), or other fields (7/140, 5%). There were no differences between groups for any of the outcomes at baseline.

**Table 1:** Sample description at baseline (n=140).

Outcome		Control group (n=70)	Intervention group (n=70)	*P* value^a^	
Age (y), mean (SD)		26.21 (5.56)	27.22 (6.96)	*.75*	
**Gender, n (%)**					*.78*
	Man	17 (24.3)	16 (22.9)		
	Woman	52 (74.3)	51 (72.8)		
	Nonbinary	1 (1.4)	2 (2.9)		
	Not specified	0 (0)	1 (1.4)		
**Highest education, n (%)**					*.78*
	Apprenticeship, vocational training, or high-school diploma	47 (67.1)	43 (61.4)		
	Higher vocational education and training	6 (8.6)	7 (10)		
	Degree at BSc^b^ level	17 (24.3)	20 (28.6)		
**Relationship status, n (%)**					*.86*
	Single	29 (41.4)	28 (40)		
	Married or in relationship	41 (58.6)	42 (60)		
**Study institute, n (%)**					*.39*
	University of Applied Science	67 (95.7)	64 (91.5)		
	University and Swiss Federal Institute of Technology ETH	3 (4.3)	4 (5.7)		
	University of Education	0 (0)	1 (1.4)		
	Others	0 (0)	1 (1.4)		
**Study subject, n (%)**					*.33*
	Applied psychology	63 (92.6)	60 (87.2)		
	Social Work	0 (0)	2 (2.9)		
	Information or technology	1 (1.5)	0 (0)		
	Economics and business	1 (1.5)	1 (1.4)		
	Pedagogy	0 (0.0)	1 (1.4)		
	Natural and earth sciences	0 (0)	1 (1.4)		
	Social sciences	3 (4.4)	3 (4.3)		
	Other	0 (0)	1 (1.4)		
**Outcomes, mean (SD)**					
	Perceived stress (PSS-10^c^)	28.79 (5.27)	28.4 (5.45)	.67	
	Depression (PHQ-9^d^)	8.16 (4.57)	7.83 (4.16)	.66	
	Anxiety (GAD-7^e^)	6.84 (4.05)	6.69 (3.77)	.81	
	Psychosomatic symptoms (PHQ-15^f^)	9.26 (4.09)	8.87 (4.39)	.59	
	Self-efficacy (GSES^g^)	29.09 (3.36)	29.21 (2.86)	.81	
	Active coping (HAPA^h^)	2.43 (0.79)	2.29 (0.85)	.31	

^a^Baseline group comparison between intervention group and waitlist control group with *t* test or chi-square test. Italicized values are statistically significant.

^b^BSc: Bachelor of Science.

^c^PSS-10: Perceived Stress Scale-10.

^d^PHQ-9: Patient Health Questionnaire-9.

^e^GAD-7: Generalized Anxiety Disorder-7.

^f^PHQ-15: Patient Health Questionnaire-15.

^g^GSES: General Self-Efficacy Scale.

^h^HAPA: health action process approach.

### Effectiveness

To evaluate the effectiveness of the intervention and to take missing data into account, a PP analysis of the time by group interaction was conducted followed by an ITT analysis. For the PP analysis (Table 2), we found evidence of a treatment effect (group by time interaction) from pre- to postintervention time points between the intervention and control groups for stress (*P*=.001; Cohen *d*=−0.60), depressive symptoms (*P*=.003; Cohen *d*=−0.50), and psychosomatic symptoms (*P*=.010; Cohen *d*=−0.36) but not for anxiety and active coping behavior.

In the ITT analysis for the unadjusted model (model 1), we found evidence of a treatment effect (group by time interaction) from pre- to postintervention time points between the intervention and control groups for stress (*P*<.001), depressive symptoms (*P*=.003), and psychosomatic symptoms (*P*=.003). No treatment effect was found for anxiety (*P*=.13) and active coping (*P*=.09).

After adjusting for the covariate self-efficacy expectancy (model 2), we found evidence of treatment effect sizes similar to model 1 (Table 3). Furthermore, there was evidence for an effect of self-efficacy expectancy on perceived stress (*P*<.001), depression (*P*<.001), anxiety (*P*<.001), and psychosomatic symptoms (*P*<.001) but not on active coping.

**Table 2:** Preintervention and postintervention means, results of the per-protocol (PP) repeated-measure ANOVA analysis, and between-group effect sizes (Cohen *d*) of primary and secondary outcomes (n=98).

Measure			Preintervention, mean (SD)	Postintervention, mean (SD)	Between-group effect sizes (intervention group vs waitlist control group after the intervention)			
					Cohen *d*^a^ (95% CI^b^)	Partial η^2^	ANOVA	
							*F* test (*df)*	*P* value
**Primary outcome**								
	**Perceived stress (PSS-10^c^)**							
		Intervention (n=42)	28.41 (5.53)	24.24 (5.93)	−0.60 (−1.01 to −0.19)	0.10	10.69 (1, 96)	.001
		Control (n=56)	28.36 (4.93)	27.61 (5.38)	—^d^	—	—	—
**Secondary outcomes**								
	**Depression (PHQ-9^e^)**							
		Intervention (n=42)	7.90 (4.24)	5.95 (3.45)	−0.50 (−0.91 to −0.10)	0.09	9.29 (1, 96)	.003
		Control (n=56)	7.86 (4.13)	7.86 (4.02)	—	—	—	—
	**Anxiety (GAD-7^f^)**							
		Intervention (n=42)	6.52 (3.69)	5.62 (3.22)	−0.29 (−0.69 to 0.11)	0.03	3.18 (1, 96)	.08
		Control (n=56)	6.41 (3.32)	6.59 (3.47)	—	—	—	—
	**Somatic symptoms (PHQ-15^g^)**							
		Intervention (n=42)	9.19 (4.81)	7.50 (3.78)	−0.36 (−0.76 to −0.04)	0.07	6.92 (1, 96)	.01
		Control (n=56)	9.07 (3.89)	9.00 (4.43)	—	—	—	—
	**Active coping (HAPA^h^)**							
		Intervention (n=42)	2.21 (0.87)	2.67 (0.75)	0.13 (−0.27 to 0.53)	0.04	3.60 (1, 96)	.06
		Control (n=56)	2.45 (0.81)	2.57 (0.78)	—	—	—	—

^a^Cohen *d* values based on means and the pooled SD of the PP analysis.

^b^95% CI of Cohen *d* (between groups, after the intervention).

^c^PSS-10: Perceived Stress Scale-10.

^d^Not applicable.

^e^PHQ-9: Patient Health Questionnaire-9.

^f^GAD-7: Generalized Anxiety Disorder-7.

^g^PHQ-15: Patient Health Questionnaire-15.

^h^HAPA: health action process approach.

**Table 3:** Results of the outcome intention-to-treat analysis (model 1), including self-efficacy as covariate (model 2), using generalized estimating equations.

Outcome		Model 1^a^		Model 2^b^	
		β estimate (SE; 95% CI)	*P* value	β estimate (SE; 95% CI)	*P* value
**Perceived stress (PSS-10^c^)**					
	Intercept	3.36 (—^d^)	—	4.18 (—)	—
	Time^e^	−0.03 (0.02; −0.08 to 0.05)	.17	−0.04 (0.02; −0.08 to 0.01)	.12
	Group^f^	−0.13 (0.03; −0.05 to 0.08)	.69	−0.01 (0.03; −0.07 to 0.05)	.75
	Treatment^g^	−0.13 (0.04; −0.20 to −0.05)	<.001	−0.12 (0.04; −0.19 to −0.04)	.001
	Self-efficacy	—	—	−0.03 (0.01; −0.04 to −0.02)	<.001
**Depression (PHQ-9^h^)**					
	Intercept	2.22 (—)	—	3.98 (—)	—
	Time	−0.01 (0.05; −0.11 to −0.09)	.83	−0.20 (0.05; −0.12 to 0.08)	.69
	Group	−0.04 (0.08; −0.20 to 0.12)	.65	−0.01 (0.08; −0.16 to 0.14)	.87
	Treatment	−0.23 (0.08; −0.38 to −0.08)	.003	−0.21 (0.07; −0.35 to −0.06)	.006
	Self-efficacy	—	—	−0.06 (0.01; −0.08 to −0.04)	<.001
**Anxiety (GAD-7^i^)**					
	Intercept	2.06 (—)	—	3.71 (—)	—
	Time	−0.00 (0.06; −0.12 to 0.12)	.99	−0.00 (0.06; −0.12 to 0.11)	.94
	Group	−0.02 (0.08; −0.18 to 0.14)	.81	−0.01 (0.08; −0.17 to 0.14)	.91
	Treatment	−0.14 (0.09; −0.31 to 0.04)	.13	−0.11 (0.09; −0.28 to 0.06)	.22
**Psychosomatic symptoms (PHQ-15^j^)**					
	Intercept	2.33 (—)	—	3.90 (—)	—
	Time	−0.01 (0.04; −0.08 to 0.61)	.77	−0.01 (0.04; −0.08 to 0.06)	.78
	Group	−0.04 (0.07; −0.18 to 0.11)	.60	−0.03 (0.07; −0.17 to 0.11)	.68
	Treatment	−0.16 (0.06; −0.27 to −0.06)	.003	−0.15 (0.06; −0.26 to −0.04)	.007
	Self-efficacy	—	—	−0.06 (0.01; −0.08 to −0.03)	<.001
**Active coping (HAPA^k^)**					
	Intercept	0.89 (—)	—	0.69 (—)	—
	Time	0.05 (0.05; −0.03 to 0.14)	.23	0.05 (0.05; −0.04 to 0.14)	.24
	Group	−0.06 (0.06; −0.17 to 0.05)	.28	−0.06 (0.06; −0.17 to 0.05)	.26
	Treatment	0.11 (0.07; −0.02 to 0.25)	.09	0.12 (0.07; −0.02 to 0.25)	.09
	Self-efficacy	—	—	0.01 (0.01; −0.01 to 0.02)	.39

^a^Model 1: unadjusted model (without covariate).

^b^Model 2: adjusted model for general self-efficacy expectancy.

^c^PSS-10: Perceived Stress Scale-10.

^d^Not applicable.

^e^Time effect represents the rate of improvement for both the intervention and waitlist control groups.

^f^Group effect represents intervention or waitlist control group.

^g^Treatment effect is represented by group and time interaction.

^h^PHQ-9: Patient Health Questionnaire-9.

^i^GAD-7: Generalized Anxiety Disorder-7.

^j^PHQ-15: Patient Health Questionnaire-15.

^k^HAPA: health action process approach.

### Exploratory

Regarding the working alliance, participants in the intervention group reported a mean working alliance score of 4.23 (SD 0.89) after the intervention. When exploring the potential influence of the working alliance on changes in outcomes from pre- to postintervention time points, we did not find evidence for correlations on any of the outcomes (Pearson correlation *r* ranging from −0.021 to 0.223). The participants rated their subjective stress expertise and goal achievement throughout the coaching program (3 times). For goal achievement, we observed a significant increase from the first to the third measurement with a large effect size (Cohen *d*=−1.07). Table 4 provides further details.

**Table 4:** Means for subscales bond, task, and goal of working alliance and results of a paired *t* test for stress expertise and goal achievement.

		Start of the intervention, mean (SD)	End of the intervention, mean (SD)	*t* test (*df*)	*P* value^a^	Cohen *d* (95% CI)
**WAI-SR^b^(n=42)**						
	Total	—^c^	4.23 (0.89)	—	—	—
	Bond	—	4.20 (1.01)	—	—	—
	Task	—	4.18 (0.82)	—	—	—
	Goal	—	4.30 (0.84)	—	—	—
Stress expertise (n=45)		7.51 (1.47)	7.64 (1.60)	0.47 (44)	.64	−0.07 (−0.36 to 0.22)
Goal achievement (n=24)		3.88 (2.54)	6.71 (2.14)	−5.24 (23)	<.001	−1.07 (−1.57 to −0.56)

^a^Within group comparison: start of intervention versus end of intervention.

^b^WAI-SR: Working Alliance Inventory with Likert scale ranging from 1 to 7.

^c^Not applicable.

### Engagement and Acceptance

In the intervention group, 60% (42/70) of the participants finished the coaching program by completing the postintervention survey (completers) and used the intervention as intended. Although the Little test indicated that values were MCAR for perceived stress (*χ^2^*_1_=0.5; *P*=.47), depression (*χ^2^*_1_=0.2; *P*=.63), anxiety (*χ^2^*_1_=2.0; *P*=.16), psychosomatic symptoms (*χ^2^*_1_=0.6; *P*=.80), and active coping (*χ^2^*_1_=0.1; *P*=.82), we conducted a dropout analysis due to the potential risk of differential attrition, particularly with significantly higher dropouts observed in the intervention group [94]. The analysis revealed no significant differences in outcomes (eg, stress and depression) or demographics (ie, gender and age) between completers and dropouts.

Overall, 45% (19/42) of the completers worked through all 13 sessions, played a mean of 86.52 (SD 120.54) minutes of relaxation audios, and received a mean of 115.88 (SD 5.06) reminders; Table 5 provides further information. On average, MISHA sent 400 (SD 205.61) SMS text messages and participants answered a mean of 297.54 (SD 169.80) SMS text messages, resulting in an average engagement ratio of 74.3%.

The participants in the intervention group (42/70, 60%) rated the subscale information highest, with a mean of 4.26 (SD 0.46), followed by engagement (mean 3.42, SD 0.70), perceived impact (mean 3.35, SD 0.87), and subjective quality of the app (mean 2.99, SD 0.87). Regarding engagement, individual customization was rated lowest with a mean of 2.71 (SD 0.84), while the target group fit was perceived as high (mean 3.95, SD 0.90). The participants liked the visual information of the CA and rated it high regarding correctness, clarity, and logic (mean 4.45, SD 0.55). Only a few participants (2/42, 2%) showed a high willingness to pay for the app (mean 2.10, SD 0.91) or anticipated high future use (mean 2.98, SD 1.05). The recommendation of the app to others was good, with a mean of 3.43 (SD 1.19) within the subjective app quality scale.

**Table 5:** Indicators of engagement: intended use, session completion, relaxation, and reminders.

Indicators of engagement	Values, n (%)	Relaxation applied, mean (SD)	Reminders received, mean (SD)^a^
Completers (intended use^b^)	42 (100)	86.52 (120.54)	15.88 (5.06)
Completed all sessions	19 (45)	97.11 (71.43)	14.89 (5.75)
Stopped interacting after session 12	3 (7)	299.33 (377.33)	17.67 (1.15)
Stopped interacting after session 11	3 (7)	29.00 (26.91)	17.67 (0.58)
Stopped interacting after session 10	1 (2)	12.00 (0.0)	27.00 (0.0)
Stopped interacting after session 9	2 (5)	46.00 (26.87)	21.00 (1.41)
Stopped interacting after session 8	4 (10)	53.50 (42.45)	16.00 (3.92)
Stopped interacting after session 7	3 (7)	89.33 (82.25)	18.00 (1.73)
Stopped interacting after session 6	3 (7)	56.00 (73.53)	17.00 (1.0)
Stopped interacting after session 5	1 (2)	26.00 (0.0)	14.00 (0.0)
Stopped interacting after session 4	1 (2)	16.00 (0.0)	9.00 (0.0)
Stopped interacting after session 3	2 (5)	4.00 (4.24)	8.50 (2.12)
Stopped interacting after session 2	0	—^c^	—
Stopped interacting after session 1	0	—	—

^a^Reminders in case of inactivity during sessions.

^b^Intended use is defined by completing the postintervention survey, regardless of number of sessions that were completed.

^c^Not applicable.

### Qualitative Feedback

**Figure 3:**
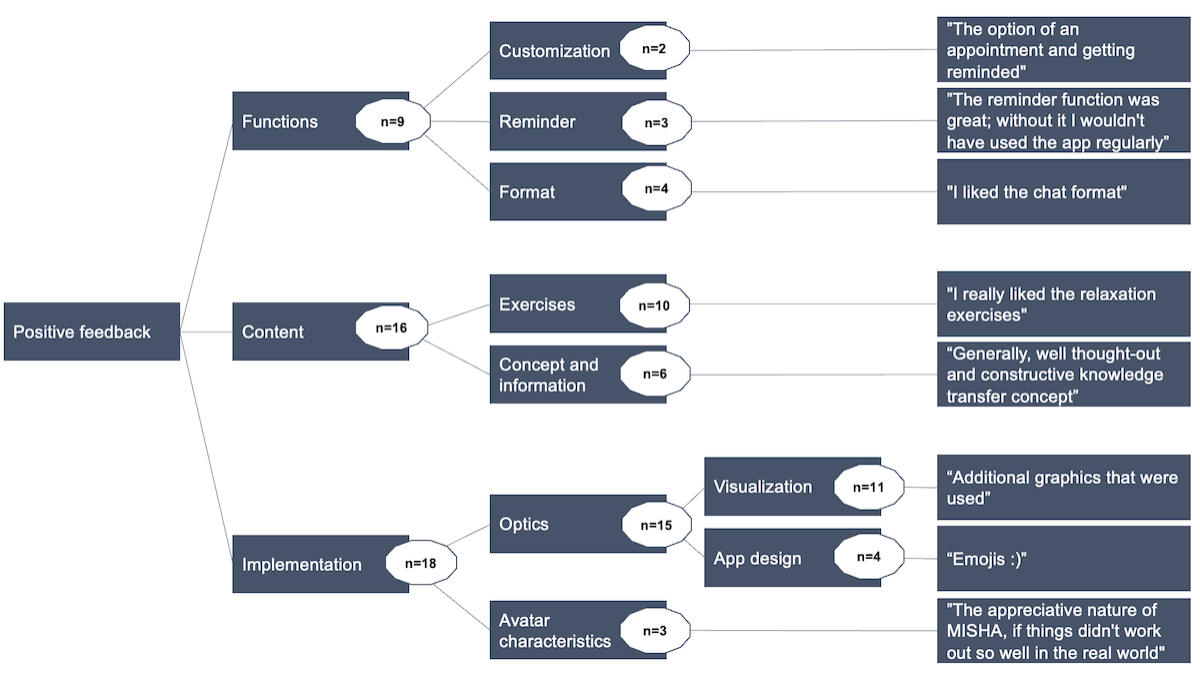
Thematic map of positive participant feedback.

**Figure 4:**
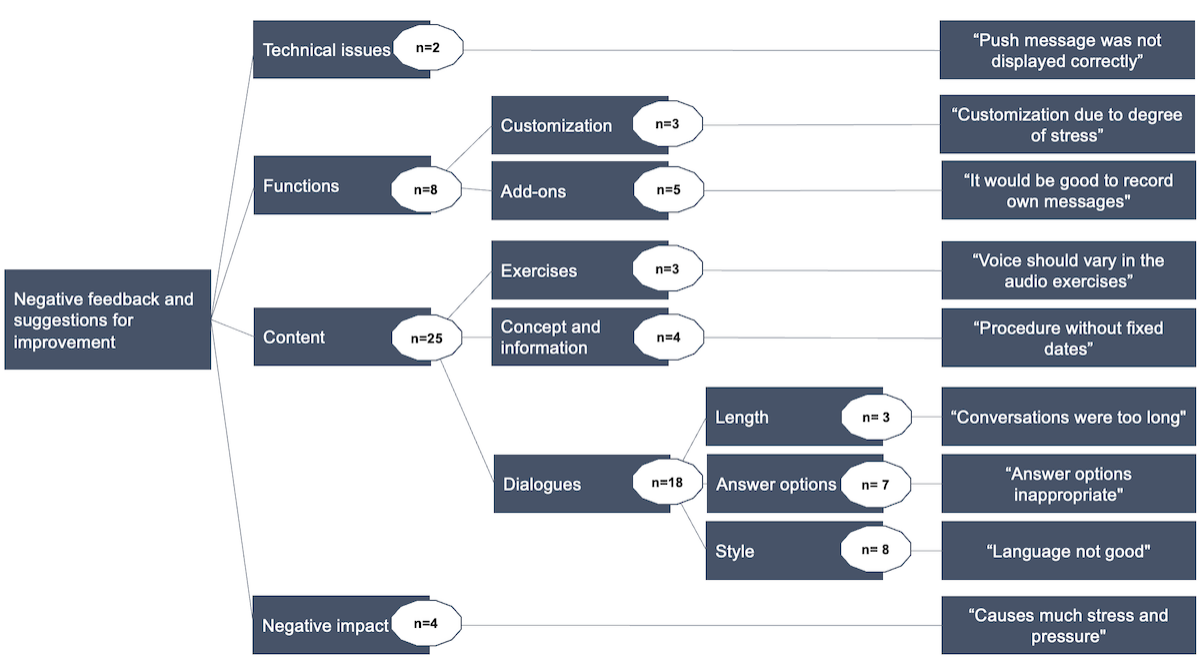
Thematic map of negative participant feedback.

The participants in the intervention group had the opportunity to provide free-text responses regarding their positive feedback on the CA intervention (Figure 3) and suggestions for improvement (Figure 4). The number of responses is displayed within the circles.

## Discussion

### Principal Findings

This study aimed to describe the development and evaluation of the effectiveness of the MISHA app, a rule-based, CA-delivered stress management coaching intervention specifically tailored to the living environment of students. We described the MISHA app’s evidence-based design and systematic evaluation. In both the PP and ITT analyses, we found evidence of decreased stress levels among participants in the intervention group compared to those in the control group, with a medium to large between-group effect (PP: Cohen *d*=−0.60). In addition, we observed evidence of a reduction in depressive symptoms with a medium to large effect (Cohen *d*=−0.50) as well as in psychosomatic symptoms with a small to medium effect (Cohen *d*=−0.36), while anxiety and active coping did not change. In the ITT analyses, a weak relation was found between self-efficacy and perceived stress, depression, anxiety, and psychosomatic symptoms, while the treatment effect persisted for stress, depression, and psychosomatic symptoms.

Our findings are consistent with other studies evaluating CA effectiveness in nonclinical populations. For instance, a study on CA Shim [95] among young adults with stress, despite a small sample size, reported stress reduction and improved psychological well-being, mirroring our results. Another study by Maciejewski and Smoktunowicz [52] assessed Stressbot, a 7-day messenger CA intervention aimed at enhancing coping self-efficacy among university students. Initial results showed reduced stress levels and improved self-efficacy postintervention. A large single-arm study evaluated Viki, an instant-messenger platform-based intervention [96], and found reduced stress and depressive symptoms. However, unlike our study, they reported a significant decrease in anxiety. In our study, the concurrent COVID-19 pandemic situation or upcoming examinations may have triggered increased uncertainties and fears. In a study involving CA Atena [53], the overall reduction in anxiety and stress levels may not have been substantial; however, the intervention showed promise in supporting individuals with high stress levels during the COVID-19 pandemic. Another study evaluated an AI-driven CA with the aim of reducing depression in university students by reflecting on their emotions, thoughts, and behavior [54]. The authors found reduced levels of depression and anxiety in the intervention group.

With its strong focus on goal setting, a crucial element in coaching [97], and being based on a behavior change model [74], the MISHA coaching intervention appears to effectively help students manage their stress. Toward the end of the coaching program, participants significantly rated their goal achievement higher with a large effect (Cohen *d*=−1.07), indicating the intervention’s effectiveness in this regard. However, some participants expressed a desire for customization options, particularly regarding stress levels.

Regarding evidence from mHealth interventions for students, a study by Yang et al [30] found positive effects on stress and overall well-being in a 30-day app-based intervention on stress management through mindfulness meditation among medical students. A systematic review confirmed that digital interventions for the enhancement of mental well-being among college students can be effective in improving depression, anxiety, and mental well-being [98].

Given the mixed findings regarding the impact of self-efficacy expectancy on stress interventions targeting students [75-77], we explored whether self-efficacy was related to perceived stress. We found only a weak relation, while the treatment effectiveness remained unchanged. Therefore, in this study, self-efficacy does not seem to have influenced the treatment’s effectiveness in reducing stress.

In line with other studies [30,99,100], participants formed a working alliance with CA MISHA. Qualitative analyses revealed participants’ appreciation for MISHA’s supportive nature, especially during challenging moments. Most participants enjoyed interacting with MISHA, found the information provided appropriate, and expressed increased intention to change their behavior related to stress. Some desired additional features (eg, voice recording), found answer options or language style to be inappropriate, and disliked the lengthy dialogues. The various exercises, reminders, and visualizations were perceived as positive, and the constructive knowledge transfer was appreciated. In summary, it appears that a CA could be a well-accepted medium for stress prevention measures among students.

### Limitations

This study has several limitations. First, despite statistically significant findings, it is essential to recognize that the absolute improvement in perceived stress, depressive symptoms, and psychosomatic symptoms was small. However, these improvements may still hold clinical relevance, and students experiencing even slight relief from perceived stress can benefit from CA-based coaching. Medium effect sizes indicate practical significance but may not always translate into substantial clinical change, and results should be interpreted with caution and in light of the context. Furthermore, all participants were self-selected, which limits the generalizability of our findings and introduces the potential for self-selection bias. Participants may have a particular interest in the subject and, therefore, cannot be considered a representative sample. It is important to note that their preexisting characteristics may differ from those in the broader population, and caution should be exercised when generalizing these findings to a wider context. Furthermore, this study is based on a convenience sample and should not be considered representative of all students. In particular, our sample, with most studying psychology (123/140, 87.9%) and predominantly woman participants (103/140, 73.6%), does not accurately reflect the student population in Switzerland, which shows an approximately even gender distribution (53% woman) [101]. Therefore, questions remain regarding the accessibility of the intervention to individuals who may not have an interest in psychological content and whether men and women can be equally reached by a mindfulness-focused chatbot such as MISHA.

Second, regarding engagement, we have analyzed use data from the intervention group, including completion rates, session completion, SMS text message response rate, reminders, and use of media player for relaxation. These objective measures offer valuable insights into participants’ interactions with the coaching program and help ensure the robustness of our findings. However, it is difficult to measure how devoted participants were when using the app. To date, there is no consensus on measuring engagement in digital interventions [81]. According to Perski et al [102], engagement can be defined as a multidimensional construct that can be measured using self-reported outcomes, use data, or even psychophysical parameters. Future research should assess participants’ time and motivation for offline engagement with exercises, while considering aspects of attention, interest, and emotions. Furthermore, in-depth use data should be gathered to assess the association between engagement, effectiveness, and optimal intended use.

Third, in this study, participants established a working alliance with the CA. However, it is important to acknowledge that CAs lack humanlike empathy or emotions [103]. They may struggle to understand the nuances of human language and lack the emotional intelligence and personal experience of a human, even if they can express empathy-like utterances. A recent study demonstrated that human-AI collaboration outperformed human-to-human collaboration, leading to a 19.6% increase in empathy in peer-to-peer text-based mental health support conversations [104]. While AI can mimic empathy and generate appropriate responses in text-based conversations, it is important to remember that these are still artificial constructs.

Fourth, various technical limitations need to be listed. At the beginning of the intervention, there were technical difficulties related to the audio files of the relaxation exercises. Some exercises could not be played. In addition, several participants indicated that the app was not updating properly; however, this issue was resolved within a few days. Furthermore, there was a 2-day interruption at the beginning because a technical adjustment had to be made to ensure that the system could recognize completed sessions. It remains unclear whether these technical issues led to more dropouts, frustration, or nonuse of the exercises. Notably, the recording of the minutes of listened audio files did not function flawlessly. While audio minutes were measured, they must be interpreted with great caution due to uncertainty in measurement. In addition, if the display of push notifications on the mobile phone was not set as the default, some SMS text messages were displayed without text. The number of people for whom this was the case and whether it negatively affected adherence cannot be conclusively determined. Any reported bugs in MISHA were addressed by a member of the study team within a 24-hour timeframe. There were no reported instances of server downtime.

Fifth, it is important to recognize the potential for improvements to enhance interaction in MISHA. The nature of the current CA is rule based: while allowing for evidence-based program development, the flexibility of interaction is limited by predefined answer options. While participants appreciated various aspects such as visualization, reminders, or exercises, personalized input via text input was missing, and some answer options were perceived as inappropriate. AI-based technology such as LLMs or natural language processing could be considered to improve text processing in MISHA. Natural language processing and LLM enable the CA to interpret user inputs more dynamically with increased natural interactions [105,106]. AI-based CAs are increasingly applied in health care to provide education and disease management. The literature on AI-based CAs indicates high overall performance and satisfactory user experience, high engagement, and positive health-related outcomes [107]. However, to date, CA interventions in the field of mental health are almost entirely rule based [108]. Ethical considerations concerning AI technology should be addressed to mitigate potential misjudgments and risks. Research highlights the critical issue of inadequate transparency in data input and algorithms, undermining the reliability and validity of results [107,109]. Currently, both rule-based and LLM-based CAs are suitable for administering script-based interventions such as CBT elements, including psychoeducation, goal setting, or reflective tasks. While in the future, LLM-based interventions may be able to deliver more complex interventions in the field of psychology, it is crucial to consider the potential risk and limitations of implementing these technologies [110].

Sixth, it is important to acknowledge the possibility of a digital placebo effect [111]. In an unblinded trial, participants might attribute their improvements to the mere use of an mHealth intervention rather than its specific components. Expectations and engagement could introduce positive bias into the outcomes. Future research should carefully plan control conditions, which might include active control groups or sham interventions [111].

### Conclusions

This paper outlines the evidence-based development of MISHA, a scalable coaching intervention specifically designed for students in their everyday life. The results of this study confirmed that CA-based coaching can be successfully delivered and is effective in reducing stress in students. It could not be confirmed that self-efficacy is related to the treatment effect. The establishment of a strong working alliance between participants and the CA, along with their perceived goal achievement, further reinforces the potential effectiveness of this intervention. Future research should involve students from diverse academic backgrounds, analyze effectiveness over time, incorporate active control groups, and improve user interaction. Overall, providing psychoeducation on stress, coupled with relaxation techniques, seems to empower students with effective tools and strategies for stress reduction.

## Appendix

Multimedia Appendix 1 Overview coaching content.

Multimedia Appendix 2 Reminder escalation.

Multimedia Appendix 3 Outcome and time points.

Multimedia Appendix 4 CONSORT-eHEALTH checklist (V 1.6.1).

## Abbreviations

AI: artificial intelligence
CA: conversational agent
CBT: cognitive behavioral therapy
CONSORT: Consolidated Standards of Reporting Trials
CONSORT-EHEALTH: Consolidated Standards of Reporting Trials of Electronic and Mobile Health Applications and Online Telehealth
GEE: generalized estimating equation
HAPA: health action process approach
ITT: intention-to-treat
LLM: large language model
MCAR: missing completely at random
mHealth: mobile Health
PHQ-9: Patient Health Questionnaire-9
PP: per-protocol
T1: time point 1
T2: time point 2
uMARS: user version of the Mobile App Rating Scale
