# Supplementary material for: A Chatbot-Delivered Stress Management Coaching for Students (MISHA App): Pilot Randomized Controlled Trial
Source: JMIR Mhealth Uhealth. 2024 Jun 26;12:e54945. doi: 10.2196/54945 (PMC11237786; doi:10.2196/54945)
Supplement: Multimedia Appendix 1 [file mhealth_v12i1e54945_app1.pdf]

Appendix 1.

Session structure

The 12 sessions are structured as follows: A) behavior change elements such as goal setting and guided self-reflection (blue colored); B) instructions and information about relaxation techniques and mindfulness (purple colored); C) psychoeducation and basic information regarding stress and its symptoms (green colored); D) student specific topics such as exam anxiety, learning techniques or time management (green colored).

All sessions are interconnected and are processed consecutively. At the beginning of coaching, participants are asked to set a personal SMART goal, which is subsequently assessed three times throughout the program. Within a session, participants have the option to delve deeper into a topic or focus on the most important or relevant content. After completion of a session, participants can set an appointment for the next session based on their preference (in 2, 3, or 4 days).

MISHA offers an integrated audio library that provides audio files for mindfulness exercises, meditations, guided imagery, and progressive muscle relaxation. All relaxation practices are introduced and incorporated into the 12 sessions of coaching. Users have access to all audio files throughout.

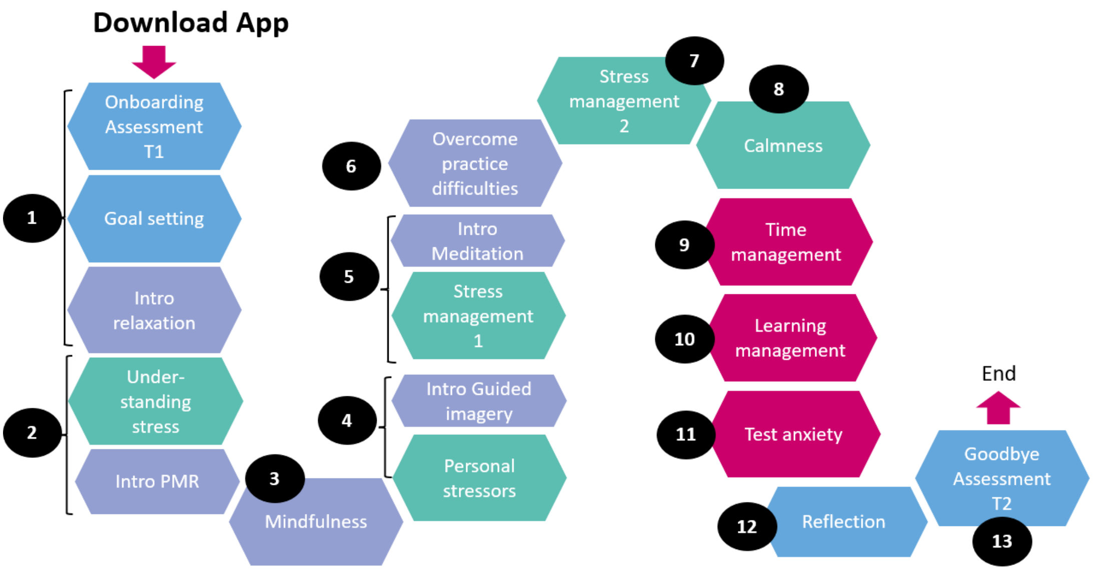

Content and sessions with MISHA.

Overview of coaching with core themes and activities[a] (translated from German).

Session	Topic	Content, guided exercises and tasks	CBT elements and techniques applied
1			
	Onboarding	• Browse<br>• Welcome greeting and presentation of coaching content<br>• Baseline survey	
	Goal setting	• SMART goal explanation and setting personal SMART goal	• Customization of treatment goal
	Intro relaxation	• Scientific background and instructions in practicing relaxation exercises<br>• Suggestion of relaxation exercise "Waterfall"	• Activity planning
2			
	Understanding stress	• Evolutionary explanation of stress<br>• Watch video "Biological Explanation of Stress"<br>• Explanation of stressors, stress enhancer, stress reaction and coping strategies<br>• Exercise: Browse stress reactions and identify own stress reaction patterns<br>• First subjective assessment of current expertise in stress-related knowledge	• Recognition of maladaptive thinking patterns as part of cognitive restructuring<br>• Data collection to evaluate efficacy of own success by participant during intervention
	Intro PMR	• Written instructions for PMR (Progressive Muscle Relaxation) and the goal of practice<br>• Encouragement to apply PMR in daily routine	• Activity planning and monitoring
3			
	Mindfulness	• Scientific evidence and its connection to stress-related problems<br>• Watch video "Meditation"<br>• Introduction of formal and informal practice<br>• Task: Define an informal mindfulness practice for this week<br>• Encouragement to practice body scan exercise	• Activity scheduling<br>• Activity planning and monitoring
4			
	Personal stressors	• Familiarize with an example of a stressful situation and discuss stress levels<br>• Familiarize with the topic of requirements and resources<br>• Watch video "Negative Thoughts"	• Identifying automatic or negative thoughts or beliefs

	Intro guided imaginary	• Exercise: Reflect own stress situation and analyze stress factors in own life<br>• Written instruction for guided imaginary<br>• Encouragement to practice guided imagery from audio library	• Activity planning and monitoring
5			
	Stress management 1	• Understand concept of instrumental stress competences<br>• Exercise: Define a situation where boundaries should be set<br>• Exercise: Identify own social network as recourse and plan strategies to cultivate social network<br>• Watch video "Calm Morning"<br>• Mental stress competences<br>• Exercise: Mental distancing with "Reframing" technique<br>• Exercise: Reflect on previously well-managed stress situations and consider how a friend would describe own competences (activating resources)<br>• Watch video "Positive Thinking"<br>• Second subjective assessment of current expertise in stress-related knowledge and progress since start of coaching	• Behavioral activation to take responsibility for own life<br>• Behavioral activation to contribute to social life<br>• Modification of maladaptive thoughts<br>• Drawing conclusion over accuracy about picture of own competences<br>• Reflection and Modification of maladaptive thoughts<br>• Reflection on own mastery and competences for modification of own beliefs
	Intro meditation	• Explanation of meditation and scientific background<br>• Encouragement to practice sitting meditation exercise from audio library	• Activity planning and monitoring
6			
	Overcome difficulties in relaxation practice	• Exercise: Check own progress in relaxation practice<br>• Information on treating common problems during relaxation practice<br>• Assess achievement of personal SMART goal no 1<br>• Encouragement to practice using the audio library	• Activity monitoring<br>• Reminding of treatment goal and reflection/evaluation of gap to goal set<br>• Activity planning and monitoring
7			
	Stress management 2	• Assess how the task from Session 3 (instrumental stress competence: setting boundaries) went (Likert Scale 1-10)<br>• Exercise: Identify personal symptoms/warning signals indicating the need for a rest<br>• Exercise: Determine personal recuperation type	• Activity monitoring<br>• Behavioral activation for self-care<br>• Activity planning

		• Task: Plan a recuperation activity for the current week in own agenda	
8			
	Calmness	• Exercise: Assess how the planning of the recuperation activity went<br>• Managing with changing process<br>• WOOP model and adaptation to personal SMART goal	• Activity monitoring<br>• Focusing customized treatment goal
9			
	Time management	• Exercise: Personal analysis of dealing with interruptions and previous experience with time management<br>• Understanding the "Eisenhower Principle", ALPEN method<br>• Task: Planning upcoming days using the ALPEN method	• Activity planning
10			
	Learning management	• Exercise: Assess the effectiveness of using ALPEN method<br>• Task: Set SMART goal for the current study semester<br>• Guidelines on creating an optimal learning environment	• Activity monitoring<br>• Activity planning
11			
	Test anxiety	• Familiarize with the "4 As" technique to address test anxiety<br>• Exercise: Assess current achievement of the second personal SMART goal and compare changes from the initial assessment	• Evaluation of current progress in order to reach personal goal
12			
	Reflection	• Watch video "Stress Management (summary and some more tips)"<br>• Exercise: "Pack Your Bag" (activating resources)<br>• Exercise: Recall personal main stressors and stress reactions and how available resources can help to cope with stressors and stress reactions<br>• Task: Apply knowledge of stress management to daily routine (keep reminders in own pocket)	• Activity planning
Between modules			
		• Anticipate difficulties of relaxation and strategies to cope with difficulties	• Activity monitoring
Outro			
		• Appreciate achievements<br>• Reflect on personal goal achievements and plan to keep the goals in mind	• Activity monitoring<br>• Evaluation of treatment and personal goal

[a]Some topics and tasks can be selected as appropriate.
