# Supplementary material for: A Chatbot-Delivered Stress Management Coaching for Students (MISHA App): Pilot Randomized Controlled Trial
Source: JMIR Mhealth Uhealth. 2024 Jun 26;12:e54945. doi: 10.2196/54945 (PMC11237786; doi:10.2196/54945)
Supplement: Multimedia Appendix 2 [file mhealth_v12i1e54945_app2.pdf]

Appendix 2

Aligning with the Health Action Process Approach (HAPA), MISHA provides users with guidance, encouragement, and reminders to help them initiate and sustain their desired actions, maintain their progress, and resume their efforts if they experience setbacks or interruptions. Every 2 days, MISHA sends reminders prompting users to engage in relaxation and imagination techniques. In case of non-response, MISHA sends reminders to motivate users to continue with the coaching according to a specific reminder escalation; the coaching discontinues at the last point where the participant provided input.

Escalation mechanism: tailored reminders (translated from German).

**Reminder for inactivity during a coaching session[a]**	
Timepoint	Examples
1 hour	Would you like to answer $coachName?
1 day	$participantName, $coachName hasn't heard from you in 1 day, please get back to him/her.
3 days	$participantName, could it be that you have owed $coachName an answer for 3 days?
**Reminder for relaxation practice**	
Every 2 days	$greeting $participantName, take your time today for your planned relaxation exercise. Or perhaps try out another one 😊<br>$greeting $participantName, change is not easy - with regular practice of relaxation you will succeed more easily. Bye 👋, $coachName
**Reminder for inactivity during data collection**	
10 min	$participantName, something seems to have come up for you....please answer the questionnaire so that the coaching can start soon!
30 min	$participantName, unfortunately you haven’t answered the questionnaire yet. Please answer all questions, only then can your data be evaluated. Thank you very much!

[a]Reminders are reset after the completion of a coaching unit (ie, the escalation process starts anew with each new coaching unit).
