# Supplementary material for: A Chatbot-Delivered Stress Management Coaching for Students (MISHA App): Pilot Randomized Controlled Trial
Source: JMIR Mhealth Uhealth. 2024 Jun 26;12:e54945. doi: 10.2196/54945 (PMC11237786; doi:10.2196/54945)
Supplement: Multimedia Appendix 3 [file mhealth_v12i1e54945_app3.pdf]

**Appendix 3**. Self-reported outcomes and timepoints.

| Time of assessments | T1[a] | Btw.[b] | T2[c] |
|---|---|---|---|
| **Screening** | | | |
| Inclusion criteria | X | | |
| Study information | X | | |
| Informed consent (digital) | X | | |
| **Demographics** | | | |
| Participant characteristics | X | | |
| **Primary outcome** | | | |
| Perceived Stress Scale PSS-10 | X | | X |
| **Secondary outcomes** | | | |
| Depressive symptoms PHQ-9 | X | | X |
| Anxiety symptoms GAD-7 | X | | X |
| Psychosomatic symptoms PHQ-15 | X | | X |
| Active coping HAPA | X | | X |
| **Predictor** | | | |
| Self-efficacy expectancy GSES | X | | |
| **Exploratory** | | | |
| Working Alliance Inventory WAI | | | X |
| Goal achievement | | X[d] | |
| Stress expertise | | X[e] | |
| **Engagement and Acceptance** | | | |
| Conversational turns answered | | X | |
| Number of completed sessions | | X | |
| Number of reminders | | X | |
| Minutes of played audios | | X | |
| Feasibility and Acceptance uMars | | | X |

[a]Baseline.
[b]Between pre-treatment and post-treatment.
[c]Primary endpoint. Intervene: Between days 24 to 54. Wait: Day 40
[d]Measured in session 1, 6, and 11.
[e]Measured during session 2, 5, and 13.
